# Impact of physical activity on maternal and neonatal outcomes among women with vaginal births—single center prospective cohort study

**DOI:** 10.3389/fmed.2025.1606471

**Published:** 2025-06-03

**Authors:** Anna Weronika Szablewska, Bartosz Zaja̧c

**Affiliations:** ^1^Department of Obstetric and Gynecological Nursing, Institute of Nursing and Midwifery, Faculty of Health Sciences with the Institute of Maritime and Tropical Medicine, Medical University of Gdańsk, Gdańsk, Poland; ^2^Laboratory of Functional Diagnostics, Central Scientific and Research Laboratory, University of Physical Education in Kraków, Kraków, Poland

**Keywords:** physical activity, pregnancy, health behavior, maternal health, infant health

## Abstract

**Background:**

Optimal levels of physical activity during pregnancy are associated with numerous health benefits for both the mother and fetus. Additionally, maternal exercise has been linked to improved cardiovascular fitness, reduced lower back pain, enhanced mental wellbeing and favorable neonatal outcomes, including a lower risk of macrosomia and improved placental function. However, both insufficient and excessive physical activity levels may have adverse effects, highlighting the need for a balanced approach.

**Objective:**

The aim of this study was to evaluate the impact of physical activity before and during pregnancy on maternal perinatal outcomes and neonatal condition. The hypothesis proposes that physical activity at the level recommended by the World Health Organization (WHO) does not negatively affect maternal or neonatal outcomes.

**Methods:**

This single-center prospective cohort study was conducted at a tertiary care hospital in northern Poland. Participants were recruited from antenatal outpatient clinics and classified based on their physical activity levels before and during pregnancy according to WHO as well as Canadian and the American College of Obstetricians and Gynecologists’ guidelines. Data collection occurred in two stages: first, through a questionnaire including socio-demographic data and the Polish version of the Get Active Questionnaire for Pregnancy, and second, by analyzing biomedical data routinely collected during childbirth. A total of 115 cases were included in the final analysis. Statistical analyses comprised logistic and linear regression model implementation, as well as the Student’s *t*-test, Welch’s *t*-test and the Mann–Whitney *U*-test, with the level of statistical significance set at *p* < 0.001.

**Results:**

No statistically significant effects of physical activity before or during pregnancy were observed on platelet count, hemoglobin levels, C-reactive protein concentrations, labor duration, BMI changes, anesthesia use, perineal trauma, or neonatal outcomes (including birth mass, length, head circumference, chest circumference, APGAR scores, and umbilical cord blood parameters).

**Conclusion:**

Physical activity before and during pregnancy does not negatively impact maternal or neonatal outcomes. These findings support current physical activity recommendations during pregnancy, emphasizing the need for further research on the mechanisms underlying hematological changes associated with exercise.

## 1 Introduction

Regular exercise contributes to reduced mortality, a decrease in the incidence of non-communicable diseases (NCDs) and improved clinical parameters (1). Leading health organizations recommend 150–300 min of moderate-intensity aerobic activity per week, supported by strength training on at least 2 days (2–5). The effectiveness of different types and modalities of physical activity in improving health has been confirmed in many meta-analyses (1, 2). However, it should be noted that physical inactivity poses a significant public health risk, increasing the likelihood of NCDs such as cardiovascular disease, type 2 diabetes and cancer, while also contributing to increased mortality and the economic burden associated with healthcare costs as well as productivity losses due to absenteeism (6, 7). Despite its many benefits, excessive physical activity may also have adverse effects, including increased risk of injury, physical overload and musculoskeletal strain, and also eating disorders associated with excessive training, such as orthorexia or Relative Energy Deficiency in Sport syndrome (8). Understanding the consequences of both insufficient and excessive physical activity is crucial for developing effective recommendations and strategies to promote a healthy lifestyle.

Optimal levels of physical activity during pregnancy are associated with numerous health benefits for both the mother and the developing fetus (9). Regular, moderate-intensity exercise has been shown to reduce the risk of gestational diabetes, hypertensive disorders and excessive gestational weight gain, which are significant risk factors for complications during pregnancy and childbirth (10–12). Moreover, physical activity contributes to improved cardiovascular fitness, reduced lower back pain and enhanced mental wellbeing, helping to alleviate symptoms of anxiety and depression commonly experienced during pregnancy (2, 13). Additionally, maternal exercise has been linked to favorable neonatal outcomes, including a lower risk of macrosomia and improved placental function, which may positively influence fetal development (14).

Conversely, a sedentary lifestyle during pregnancy can lead to adverse health effects, increasing the likelihood of excessive weight gain, insulin resistance and gestational hypertension, all of which may contribute to complications such as pre-term birth or the need for cesarean delivery (15–18). Prolonged inactivity is also associated with a higher risk of developing deep vein thrombosis and experiencing more severe postpartum recovery. Furthermore, physical inactivity may negatively impact fetal growth and development, as maternal metabolic and cardiovascular health play a crucial role in supporting optimal intrauterine conditions (10, 12, 19, 20).

While moderate exercise is beneficial, excessive physical activity—especially in high-intensity sports—can pose potential risks to both maternal and fetal health. Pregnant athletes or individuals engaging in strenuous exercise regimes may be at an increased risk of energy deficits, hormonal imbalances and impaired fetal growth due to reduced placental blood flow (12). Intense physical strain has also been associated with an increased risk of pre-term labor, musculoskeletal injuries and disruptions in menstrual cycles prior to conception, which may affect fertility and pregnancy outcomes (21). Therefore, it is essential to balance physical activity during pregnancy, ensuring that it supports maternal health without imposing unnecessary physiological stress on the mother and fetus.

Despite the well-documented benefits of physical activity during pregnancy, few studies have examined its effects on labor characteristics and neonatal outcomes in a strictly defined population of women delivering vaginally. Most existing analyses combine different modes of delivery, limiting the ability to isolate specific associations. This study aims to address this gap. Addressing the present research problem was motivated by the well-documented positive effects of physical activity on population health, including maternal and neonatal wellbeing. A key limitation of previous studies is the lack of analyses focusing exclusively on women who have given birth vaginally. Isolating this group enables more precise assessment regarding the impact of physical activity on parameters such as the duration of different labor phases, perineal trauma and the type of anesthesia used—factors that cannot be meaningfully compared in the case of cesarean delivery. Additionally, neonatal outcomes are directly influenced by the mode of delivery and its underlying causes, further supporting the need for a homogeneous study population. The aim of this study was to evaluate the impact of physical activity before and during pregnancy on maternal perinatal outcomes and neonatal condition. The proposed hypothesis is that physical activity at the level recommended by the WHO does not negatively affect maternal or neonatal outcomes.

## 2 Materials and methods

### 2.1 Study design

This single-center, prospective cohort study was conducted at a tertiary care hospital in northern Poland. Participants were recruited from among patients attending the antenatal outpatient clinic between September and December 2024. Data for the research were collected in two stages. In the first stage, participants who met the inclusion criteria were asked to complete a questionnaire consisting of self-reported questions in the form of a medical interview, provide socio-demographic data and fill out the Polish version of Get Active Questionnaire for Pregnancy (GAQ-P_*PL*_) (22). The questionnaire included questions about systemic diseases such as respiratory and cardiovascular conditions, epilepsy, diabetes, thyroid disorders, eating disorders, anemia and hypertension. Patients also provided information about the course of their current pregnancy, including whether they experienced complications such as intrauterine growth restriction, the fetus being small for gestational age, genital bleeding, pre-term pre-mature rupture of membranes, cervical insufficiency or a cervical cerclage. Additionally, patients were asked about their obstetric history, including recurrent miscarriages and pre-term births. By answering “YES” or “NO” to these questions, it was possible to identify pregnant women with contraindications to physical activity or those who avoided it due to a history of severe obstetric complications. The participants were also asked to assess their physical activity levels at two different stages: 6 months before pregnancy and up until the time of completing the questionnaire. Furthermore, the obstetric situation was taken into account, including potential contraindications to physical activity, particularly in cases of cervical shortening and placenta-related conditions. Recruiting women at this stage allowed to assess their physical activity throughout pregnancy and enabled planning for its remainder.

Participants were classified twice based on GAQ-P_*PL*_ data: first, according to their physical activity status before pregnancy—physically active (PAG_*B*_) or physically inactive (PIG_*B*_)—and then according to their status during pregnancy—physically active (PAG_*D*_) or physically inactive (PIG_*D*_). The classification was based on the WHO, Canadian guidelines (9) and American College of Obstetricians and Gynecologists recommendations for physical activity during pregnancy and basic adult activity (23)—at least 150 min of moderate-intensity or 75 min of vigorous-intensity activity per week (24). Detailed characteristics of the physical activity performed by the PAG_*B*_ and PAG_*D*_ groups are presented in Table 1. In the second stage, access was obtained to standard biomedical data concerning the participants and their newborns, which were routinely collected during childbirth. All procedures were in accordance with the principles outlined in the 1964 Declaration of Helsinki, and its subsequent amendments. Permission to conduct the research was obtained from the Bioethics Committee of the Medical University of Gdańsk (No. NKBBN/406-1/2024).

**TABLE 1:** Characteristics of physical activity among women who were physically active before and during pregnancy.

	PAG_*B*_	PAG_*D*_
**Frequency [no. of activities per week]**		
1-2	2.0%	6.7%
3-4	80.0%	60.0%
5-7	18.0%	33.3%
**Duration [min. per session]**		
<20	0.0%	0.0%
20-30	2.0%	6.7%
31-60	58.0%	63.3%
> 60	40.0%	30.0%
**Intensity**		
Low	2.0%	13.3%
Moderate	78.0%	80.0%
High	20.0%	6.7%
Type*		
Endurance	42.0%	40.0%
Strength	26.0%	26.7%
Other	32.0%	33.3%

PAG_*B*_/PAG_*D*_, physically active group before/during pregnancy.

*Type: endurance (swimming, cycling, walking, aerobic) strength (pilates, Joga, weight lifting), other (stretching, exercises preparing for childbirth).

### 2.2 Participants

A total of 230 Caucasian women were included in the study based on the following inclusion criteria: beginning of the third trimester (after the 28th *^w^*eek of pregnancy), age 18 and above, proficiency in the Polish language, no contraindications to physical activity, singleton pregnancy, absence of placenta previa and planned delivery at a hospital affiliated with the outpatient clinic from which the participants were recruited. A total of 115 cases were included in the final analysis. The reasons for excluding the remaining participants were: delivery at a different hospital (17 cases), pre-term birth (8 cases) and delivery by cesarean section (90 cases). The sample size was calculated *a priori* using the G*Power 3.1 calculator (Franz Faul, University of Kiel, Germany), with the following assumptions: two-tailed test; effect size *f*^2^ = 0.07; α (error probability) = 0.05; power (1-β error probability) = 0.80; number of predictors = 4. Furthermore, it was assumed that 50% of the observations would be lost, primarily due to pregnancies ending in cesarean section. Participants were thoroughly informed about the purpose and protocol of the study, as well as their right to withdraw from the study at any time. The participants provided written informed consent to participate in the research. The women gave their consent not only to take part in the study but also to allow access to anonymized medical data concerning both themselves and their newborns. The basic characteristics of study participants are presented in Table 2.

**TABLE 2:** Basic characteristics of the study participants.

	PAG_*B*_, *n* = 50	PIG_*B*_, *n* = 65	*p*-value	PAG_*B*_, *n* = 30	PIG_*D*_, *n* = 85	*p*-value
Age [years]	31.1 ± 4.24	31.6 ± 4.6	0.568	31.9 ± 4.7	31.2 ± 4.3	0.478
Body height [m]	1.68 ± 5.9	1.66 ± 6.8	0.387	1.68 ± 5.28	1.67 ± 6.82	0.407
Pre-pregnancy body mass [kg]	65.9 ± 12.4	66.5 ± 15.1	0.834	63.4 ± 10.0	67.5 ± 15.0	0.174
Pre-pregnancy BMI [kg × m^–2^]	23.4 ± 4.0	23.9 ± 4.9	0.584	22.5 ± 3.3	24.2 ± 4.8	0.079
Mass gain during pregnancy^∧^			0.200			0.415
Insufficient	20 (40.0) [55.5]	16 (24.6) [45.5]		16 (24.6) [45.5]	24 (28.2) [68.6]	
Adequate	14 (28.0) [40.0]	21 (32.3) [60.0]		6 (21.4) [17.6]	28 (32.9) [82.4]	
Excessive	16 (32.0) [36.4]	28 (43.1) [63.6]		11 (39.3) [25.0]	33 (38.8) [75.0]	
Education level			0.333			0.656
Primary	3 (6.0) [37.5]	5 (7.7) [62.5]		2 (7.1) [25.0]	6 (7.1) [75.0]	
Secondary	5 (10.0) [31.3]	11 (16.9) [68.7]		2 (7.1) [12.5]	14 (16.5) [87.5]	
Higher	42 (84) [46.2]	49 (75.4) [53.8]		24 (85.8) [27.0]	65 (76.4) [73.0]	
Gravidity category			0.974			0.114
Primegravida	26 (52.0) [43.3]	34 (52.3) [56.7]		18 (64.3) [31.0]	40 (47.1) [69.0]	
Multigravida	24 (48.0) [43.6]	31 (47.7) [56.4]		10 (35.7) [18.2]	45 (52.9) [81.8]	
Place of residence			0.094			0.006
Village	12 (24.0) [63.2]	7 (10.8) [36.8]		5 (17.9) [26.3]	14 (16.5) [73.7]	
City ≤ 500k inhabitants	32 (64.0) [37.6]	53 (81.5) [62.4]		16 (57.1) [19.3]	67 (78.8) [80.7]	
City > 500k inhabitants	6 (12.0) [54.5]	5 (7.7) [45.5]		7 (25.0) [63.6]	4 (4.7) [36.4]	
Economic status			0.482			0.631
Average	6 (12.0) [37.5]	10 (15.4) [62.5]		3 (10.7) [20.0]	12 (14.3) [80.0]	
Good	44 (88.0) [44.4]	55 (84.6) [55.6]		25 (89.3) [25.8]	72 (85.7) [74.2]	
Marital status			0.234			0.326
Single	9 (18.0) [32.1]	19 (29.2) [67.9]		6 (21.4) [22.2]	21 (24.7) [77.8]	
Married	39 (78.0) [45.9]	46 (70.8) [54.1]		21 (75.0) [25.0]	63 (74.1) [75.0]	
Widowed	1 (2.0) [100.0]	0 (0.0) [0.0]		0 (0.0) [0.0]	1 (1.2) [100.0]	
Divorced	1 (2.0) [100.0]	0 (0.0) [0.0]		1 (3.6) [100.0]	0 (0.0) [0.0]	

Continuous data are presented as mean ± SD, and categorical data as n with column percentage (in parentheses) and row percentage [in square brackets]. Data do not sum up to the total sample size due to missing values. PAG_*B/D*_, physically active group before/during pregnancy; PIG_*B/D*_, physically inactive group before/during pregnancy; *p*-value, probability of obtaining the observed results under the null hypothesis. ^∧^Mass gain during pregnancy based on pre-pregnancy BMI was assessed according to IOM recommendations for optimal maternal and fetal outcomes (55).

### 2.3 Maternal outcome

Data obtained from maternal medical records collected during childbirth included information on hemoglobin concentration, C-reactive protein (CRP) levels, platelet count, the duration of the first (is recognized when there are regular contractions leading to progression of labor, i.e., dilation of the cervix and descent of the baby into the birth canal) and second stages of labor (begins when the cervix is fully dilated (10 cm) and the baby is born) (25), maternal weight gain during pregnancy, the type of anesthesia used and birth-related perineal injuries.

### 2.4 Neonatal outcome

Neonatal data obtained from medical records included birth mass, birth length as well as head and chest circumferences, APGAR scores (the APGAR score—named after Dr. Virginia Apgar—assesses five key indicators of newborn health: Appearance, Pulse, Grimace, Activity, and Respiration) at 1 and 5 min (26), as well as umbilical-cord arterial blood gas analysis such as blood pH, partial pressure of carbon dioxide (pCO_2_), partial pressure of oxygen (pO_2_) and oxygen saturation (in percentages; pulse oximetry screening is performed on the infant’s right lower limb).

### 2.5 Statistical analysis

Statistical analysis was performed in the Python programming language using the following libraries: pandas, numpy, scikit-learn, statsmodels, scipy, and statsmodels.stats. To assess the effect of physical activity on dependent variables measured on nominal or ordinal scales, logistic regression analysis was applied. For dependent variables measured on a continuous scale, linear regression was employed, preceded by verification of the model’s main assumptions, including evaluation of residual normality (via histogram assessment), homoscedasticity (using the Breusch–Pagan test), and multicollinearity (using the Variance Inflation Factor, with a VIF value exceeding 5 indicating potential concern). Differences between groups were examined using the Student’s *t*-test, Welch’s *t*-test, or the Mann–Whitney *U*-test for continuous variables, depending on whether assumptions of normal distribution (verified by the Shapiro–Wilk test) and homogeneity of variances (assessed via Levene’s test) were met. For categorical variables comparisons were performed using the χ^2^-test. After Bonferroni correction, a *p*-value below 0.001 was considered statistically significant.

## 3 Results

### 3.1 Maternal outcomes

The analysis did not reveal any statistically significant associations between physical activity—either before or during pregnancy—and platelet count, hemoglobin levels, C-reactive protein concentrations, or the duration of the first and second stages of labor were observed. For platelet count, *p*-values below 0.03 were obtained for physical activity both before pregnancy and during pregnancy. Detailed numerical results are presented in Table 3.

**TABLE 3:** Effect of maternal physical activity on labor outcomes and maternal condition.

	PA_*B*_					PA_*D*_				
	β	SE	95% CI	R^2^	*p*-value	β	SE	95% CI	R^2^	*p*-value
Hemoglobin [g × dL]	0.01	0.26	–0.50 to 0.51	0.03	0.989	–0.19	0.30	–0.79 to 0.41	0.04	0.535
CRP [mg × L^–1^]	1.64	3.46	–5.23 to 8.50	0.01	0.638	5.58	3.99	–2.34 to 13.50	0.02	0.165
Platelets [10^3^ × μL^–1^]	–25.6	11.0	–47.5 to (–3.8)	0.10	0.022	–31.1	13.0	–56.9 to (–5.3)	0.11	0.019
1^st^ stage of labor [min]	–18.3	30.2	–78.3 to 41.7	0.10	0.546	–30.1	36.2	–101.9 to 41.7	0.10	0.408
2^nd^ stage of labor [min]	–5.60	6.58	–18.6 to 7.4	0.25	0.396	8.03	7.79	–7.41 to 23.47	0.27	0.305

The reference value for the variable *physical activity before/during pregnancy* is *no activity* (coded as 0). The model was adjusted for gravidity category, hypertension, and gestational diabetes. The amount of missing data for each dataset did not exceed 8%. PA_*B/D*_, physical activity before/during pregnancy; β, regression coefficient; SE, standard error; 95% CI, 95% confidence interval; CRP, C-reactive protein; R^2^, coefficient of determination; *p*-value, probability of obtaining the observed results under the null hypothesis.

The analysis did not reveal any statistically significant associations between physical activity—either before or during pregnancy—and the risk of insufficient or excessive BMI gain, type of anesthesia used during labor or the occurrence of perineal trauma. Detailed numerical results are presented in Table 4.

**TABLE 4:** Effect of maternal physical activity on BMI gain, type of anesthesia and perineal trauma.

	PA_*B*_			PA_*D*_		
	Odds ratio	95% CI	*p*-value	Odds ratio	95% CI	*p*-value
**Weight gain during pregnancy^∧^**						
Insufficient	2.05	0.92–4.55	0.079	1.61	0.65–3.99	0.299
Excessive	0.62	0.29–1.34	0.227	0.98	0.41–2.38	0.970
Adequate	Ref.	Ref.	Ref.	Ref.	Ref.	Ref.
**Type of anesthesia**						
Non-pharmacological	2.74	0.48–15.6	0.256	0.14	0.00–9.25	0.359
Pharmacological	1.19	0.39–3.63	0.765	0.76	0.21–2.64	0.671
No anesthesia	Ref.	Ref.	Ref.	Ref.	Ref.	Ref.
**Presence of perineal traumas**						
Perineal trauma present	1.12	0.52–2.44	0.767	1.00	0.41–2.47	0.992
Perineal trauma absent	Ref.	Ref.	Ref.	Ref.	Ref.	Ref.

The reference value for the variable *physical activity before/during pregnancy* is *no activity* (coded as 0). The model was adjusted for the mother’s gravidity category. The amount of missing data for each dataset did not exceed 8%. PA_*B/D*_, physical activity before/during pregnancy; 95% CI, 95% confidence interval*; p*-value, probability of obtaining the observed results under the null hypothesis. ^∧^Mass gain during pregnancy based on pre-pregnancy BMI was assessed according to IOM recommendations for optimal maternal and fetal outcomes (25).

### 3.2 Neonatal outcomes

The analysis did not show any statistically significant effects of physical activity—neither before nor during pregnancy—on neonatal outcomes, including birth mass, birth length, head and chest circumferences, APGAR scores at 1 and 5 min, umbilical cord blood pH, pCO_2_, pO_2_, or oxygen saturation. Detailed numerical results are presented in Table 5.

**TABLE 5:** Effect of maternal physical activity on neonatal health indicators.

	PA_*B*_					PA_*D*_				
	β	SE	95% CI	R^2^	*p*-value	β	SE	95% CI	R^2^	*p*-value
Birth mass [g]	–92.8	114.3	–319.4 to 133.7	0.01	0.418	105.6	135.2	–162.4 to 373.7	0.01	0.436
Birth length [cm]	–1.31	0.77	–2.83 to 0.22	0.03	0.093	0.91	0.92	–0.91 to 2.73	0.02	0.322
HC [cm]	–0.34	0.43	–1.20 to 0.52	0.03	0.436	0.79	0.51	–0.22 to 1.79	0.05	0.122
CC [cm]	–0.64	0.54	–1.71 to 0.42	0.05	0.235	0.72	0.63	–0.53 to 1.98	0.05	0.255
APGAR^1min^ [0-10 pt.]	–0.13	0.30	–0.72 to 0.45	0.01	0.651	0.46	–0.35	–0.22 to 1.15	0.03	0.183
APGAR^5min^ [0-10 pt.]	–0.16	0.20	–0.55 to 0.24	0.02	0.425	0.19	0.24	–0.28 to 0.65	0.05	0.430
Cord blood pH	0.14	0.01	–0.03 to 0.04	0.14	0.798	0.01	0.02	–0.03 to 0.06	0.14	0.589
pCO_2_ [mmHg]	0.17	2.27	–4.32 to 4.66	0.09	0.941	2.12	2.70	–4.32 to 4.66	0.10	0.434
pO_2_ [mmHg]	0.54	2.50	–4.42 to 5.50	0.04	0.829	0.61	2.96	–5.26 to 6.49	0.04	0.837
Saturation [%]	0.10	0.34	–0.58 to 0.77	0.02	0.781	0.06	0.40	–0.73 to 0.84	0.02	0.881

The reference value for the variable *physical activity before/during pregnancy* is *no activity* (coded as 0). The model was adjusted for gravidity category, hypertension, and gestational diabetes. The amount of missing data for each dataset did not exceed 8%. PA_*B/D*_, physical activity before/during pregnancy; HC, head circumference; CC, chest circumference; β, regression coefficient; SE, standard error; 95% CI, 95% confidence interval; R^2^, coefficient of determination; *p*-value, probability of obtaining the observed results under the null hypothesis; pCO_2_/O_2_, partial pressure of carbon dioxide/oxygen.

## 4 Discussion

The aim of this study was to assess the impact of physical activity before and during pregnancy on maternal perinatal outcomes and neonatal condition. Given the well-established benefits of physical activity for overall health, including maternal and neonatal wellbeing, in our research, we sought to address a gap in the literature: the lack of studies focusing exclusively on women who had given birth vaginally. By isolating this group, we were able to provide a more precise evaluation of labor parameters such as the duration of its different phases, perineal trauma and the type of anesthesia used—factors that cannot be meaningfully analyzed in the context of cesarean deliveries. Our findings support the hypothesis that engaging in physical activity at levels recommended by the WHO does not negatively impact maternal or neonatal outcomes. These results have significant clinical implications, reinforcing the recommendation that pregnant women can safely maintain an active lifestyle without fear of compromising their health or that of their newborns.

In our study, we observed a lower platelet concentration in physically active women both before and during pregnancy compared to physically inactive women. While this difference reached statistical significance (*p* < 0.03), it did not remain significant after applying the Bonferroni correction for multiple comparisons. Therefore, this finding should be interpreted cautiously. Nonetheless, the observed trend may suggest a potential physiological adaptation to regular physical activity, which could influence hematological parameters during pregnancy. This aligns with previous research indicating that physical activity may affect platelet function and activation (27, 28). While acute, vigorous exercise has been shown to transiently increase platelet activation (29), habitual moderate physical activity appears to have a regulatory effect, reducing platelet reactivity and contributing to cardiovascular protection (30). This is particularly relevant in pregnancy, during which physiological changes lead to an increase in platelet count, especially in the second trimester, as a protective mechanism against postpartum hemorrhage (31). However, pregnancy is also associated with a heightened risk of thromboembolic events. The observed decrease in platelet count among physically active women insinuates a potential regulatory effect of exercise, which may help maintain hemostatic balance and reduce thrombotic risk during pregnancy. In future studies, the implications of these findings should be further explored, particularly in relation to pregnancy-related thromboembolic complications. In contrast, no significant effects of physical activity were observed on hemoglobin levels, C-reactive protein concentrations or the duration of the first and second stages of labor. The absence of a relationship between hemoglobin levels and physical activity is consistent with previous studies, in which inconclusive results have been reported in this regard. Hemoglobin levels during pregnancy are influenced by multiple factors, including hemodilution due to plasma volume expansion, iron status, dietary intake and supplementation (32, 33). While physical activity has been suggested to enhance the absorption and utilization of iron, its impact on hemoglobin levels may be subtle and overshadowed by these other physiological and nutritional variables (34). Similarly, CRP levels did not differ significantly between physically active and inactive women. CRP is a well-established marker of systemic inflammation, and while in some studies it is suggested that regular physical activity may lower CRP levels by reducing chronic inflammation, the effect is often more pronounced in populations with preexisting metabolic or inflammatory conditions (35, 36). In healthy, pregnant women, the natural immunological adaptations of pregnancy, alongside individual differences in baseline inflammatory status, may attenuate the potential impact of physical activity on CRP levels (35).

Regarding labor duration, the lack of significant differences between active and inactive women may be explained by the multifactorial nature of labor progression. While in some studies shorter labor durations have been reported among physically active women, in others, no significant effects have been found (35). Additionally, factors such as individual variability in cervical ripening and uterine contractility may have had stronger impact on labor duration than physical activity alone. The heterogeneity in exercise type, intensity and timing across different studies may also contribute to the inconsistent findings in the literature (35). Further research, particularly stratified analyses based on parity and exercise characteristics, is needed to clarify the relationship between physical activity and labor duration.

Our analysis did not reveal any statistically significant correlations between physical activity—neither before nor during pregnancy—and the risk of insufficient or excessive BMI gain, the type of anesthesia used during labor or the occurrence of perineal trauma. These findings are in contrast with those observed in some previous studies, in which it has been suggested that physically active women tend to have better weight control during pregnancy due to greater health awareness and established lifestyle habits. Regular physical activity has been associated with a lower risk of excessive gestational weight gain, potentially due to its role in regulating energy balance and metabolism (37). However, the lack of a significant effect in our study may be attributed to multiple factors. First of all, weight gain during pregnancy is influenced by a complex interplay of genetic, metabolic and behavioral factors, including dietary habits, hormonal changes and individual metabolic responses (38–40). It is possible that the variability in exercise type, duration and intensity among our study participants, as well as differences in dietary intake and metabolic adaptations, may have masked any potential effects of physical activity on weight gain.

Similarly, no significant relationships were found between physical activity and the type of anesthesia used during labor. While physical activity is known to improve pain tolerance and psychological resilience, labor pain is a highly subjective experience influenced by numerous factors, including individual pain thresholds, fetal positioning, labor duration and maternal stress levels (41, 42). Epidural analgesia remains the most widely used form of pain relief during labor, and conflicting results have been reported in previous studies regarding its association with prenatal physical activity. In some studies, it has been suggested that physically active women may have a lower demand for pharmacological pain relief due to enhanced pain coping mechanisms (43–45), while in others, including meta-analyses, no significant differences were in epidural use between active and inactive women (46). Our findings align with the latter, suggesting that factors beyond physical activity, such as labor management protocols, maternal preferences and access to pain relief options, play a more decisive role in determining anesthesia use during childbirth (46).

Our analysis did not show any statistically significant effects of physical activity—neither before nor during pregnancy—on neonatal outcomes, including birth mass, birth length, head and chest circumferences, APGAR scores at 1 and 5 min, umbilical cord blood pH, pCO_2_, pO_2_ or oxygen saturation. These findings reinforce the notion that maternal physical activity does not pose a risk to the neonate and supports existing evidence on the safety of prenatal exercise. In previous studies, it has been suggested that certain neonatal parameters, such as oxygen saturation, may be improved in infants born to physically active mothers (34). However, while some benefits have been indicated, our findings align with broader research showing that key indicators of neonatal wellbeing—such as APGAR scores and umbilical cord blood gasometry—are not significantly influenced by maternal physical activity. The lack of an observed effect suggests that while physical activity during pregnancy may support maternal health and reduce pregnancy-related complications, it does not necessarily enhance neonatal outcomes beyond physiological norms.

The absence of a significant association between maternal physical activity and newborn birth mass aligns with previous research, producing mixed results. In some studies, a slight reduction has been suggested in birth mass among newborns of physically active women (37, 38, 47, 48), possibly due to improved maternal glucose metabolism and better control of gestational weight gain. However, in other studies (14), including ours, no significant impact has been found, indicating that physical activity within recommended levels does not adversely affect fetal growth but also does not necessarily enhance it. Given that birth mass is influenced by multiple factors—including maternal nutrition, genetics, placental function and gestational age—it is probable that any potential effects of physical activity are masked by these stronger determinants (49). Similarly, in our study, no significant impact of physical activity was observed with regard to neonatal head and chest circumferences. These parameters are key indicators of fetal development and intrauterine growth patterns, and their stability suggests that prenatal exercise does not compromise fetal development in terms of skeletal and soft tissue growth.

An important aspect of our study is the analysis of umbilical cord blood gasometry, which provides objective insights into the newborn’s respiratory and metabolic status at birth. While we found no significant differences in umbilical cord blood pH, pCO_2_, pO_2_ or oxygen saturation, it is worth noting that studies specifically focused on arterial cord blood gas analysis in relation to maternal physical activity are limited (50, 51). Our findings contribute novel data to this underexplored area, emphasizing the need for further research to clarify whether maternal physical activity influences fetal oxygenation and acid-base balance at birth. Considered as a whole, our results reinforce current recommendations promoting physical activity during pregnancy as a safe practice that does not compromise neonatal outcomes. However, given the limited number of studies in which neonatal blood gas parameters are analyzed in this context, in future research, the potential mechanisms through which maternal physical activity may influence fetal oxygenation and metabolic adaptation at birth should be investigated.

Additionally, our findings indicate that place of residence was significantly associated with physical activity levels during pregnancy (*p* = 0.006). A more detailed breakdown shows that women living in rural areas were proportionally less physically active compared to those in urban settings. This may reflect differences in access to infrastructure for physical activity (e.g., parks, gyms), cultural norms, or health education resources available in larger cities. These findings are consistent with previous research suggesting urban residents may have greater opportunities and support for maintaining physical activity during pregnancy (14, 52–54). However, due to the uneven distribution of participants across residence categories, further research is needed to confirm and expand on this association.

This study offers several notable strengths. By including only women who underwent vaginal delivery, we minimized clinical heterogeneity and improved the internal validity of the study by excluding confounding factors associated with cesarean section, such as surgical trauma, anesthesia, and varying medical indications for operative delivery. Physical activity levels were categorized based on internationally recognized guidelines, including those from the World Health Organization (WHO), the American College of Obstetricians and Gynecologists (ACOG), and Canadian recommendations. Additionally, the use of the validated Polish version of the “Get Active Questionnaire for Pregnancy” (GAQ-PPL) enhanced the reliability of the self-reported data.

Nevertheless, the study is not without limitations. First, its observational design precludes causal inferences. While associations between physical activity and maternal and neonatal outcomes were identified, unmeasured confounding variables—such as dietary patterns or psychosocial factors—could have influenced the results. Second, despite using a validated tool, physical activity was assessed via self-report, which is subject to recall bias and potential over- or underestimation of activity levels. Finally, the focus on women with vaginal delivery improves internal validity but limits the generalizability of findings to the broader population, including those undergoing cesarean sections.

To build upon the current findings, future research should consider conducting multicenter studies with larger and more diverse populations to enhance external validity. Incorporating wearable devices could provide precise, continuous monitoring of physical activity and allow for assessment of exercise intensity and patterns across pregnancy trimesters. Moreover, it would be valuable to explore the biological mechanisms that may underlie the observed associations, such as the influence of exercise on inflammatory markers, hematologic profiles, and hormonal changes during pregnancy. Further studies should also include women with both vaginal and cesarean deliveries to compare how mode of birth may moderate the impact of physical activity on maternal and neonatal outcomes.

The findings of this study may have significant implications for clinical practice, providing evidence on the safety of physical activity during pregnancy. By reinforcing the absence of adverse effects, these results could encourage pregnant women to engage in regular exercise, alleviating concerns about potential health risks for both themselves and their newborns. Our findings suggest that engaging in physical activity before and during pregnancy, within the recommended guidelines set by the WHO, is safe and does not have negative effects on maternal or neonatal outcomes. This provides important reassurance to healthcare providers who may encounter concerns from expectant mothers regarding the safety of exercise during pregnancy. Furthermore, by identifying the potential benefits of regular physical activity—such as a reduction in platelet levels—it offers a foundation for promoting exercise as a protective factor against complications such as thrombosis, which can be of particular concern during pregnancy. Given that physical activity does not negatively affect neonatal parameters, such as birth mass, APGAR scores or cord blood gases, these results encourage the promotion of physical exercise as part of prenatal care. Healthcare providers can advise pregnant women to continue or adopt physical activity routines, knowing that the practice will not put their pregnancy at risk. Additionally, the importance of fostering a healthy lifestyle prior to pregnancy is highlighted in the study, as women who were active before conception may already have better health practices in place, which may contribute to maintaining healthy weight gain during pregnancy and optimal maternal health outcomes. This study also allows to reinforce the need for individualized exercise recommendations during pregnancy, considering the varying responses among women. These findings support the incorporation of structured physical activity counseling into routine prenatal visits, and suggest that healthcare professionals should proactively reassure and encourage pregnant women to maintain or initiate moderate-intensity exercise in accordance with WHO guidelines.

## 5 Conclusion

The findings of this study suggest that engaging in physical activity in accordance with WHO guidelines before and during pregnancy is not associated with adverse maternal or neonatal health outcomes. While no statistically significant negative effects were observed, the study was not powered to detect small differences, and therefore conclusions about safety should be interpreted with caution. A trend toward lower platelet counts in physically active women was noted, although this association did not remain statistically significant after Bonferroni correction. This finding may still be of clinical relevance and warrants further investigation in larger samples. Physical activity may have beneficial effects on certain maternal parameters, such as cardiovascular function and thrombotic risk, but these mechanisms require confirmation through future research.

Although no significant impact on neonatal outcomes was observed, these results do not provide definitive evidence of equivalence. Future studies with larger cohorts and objective measurement of physical activity are needed to better understand its effects on both maternal and neonatal outcomes. Overall, the findings support the continued recommendation of physical activity during pregnancy, but underscore the need for further studies to confirm its safety and benefits across different subpopulations.

## Data Availability

The datasets presented in this study can be found in online repositories. The names of the repository/repositories and accession number(s) can be found at: https://ppm.gumed.edu.pl/info/researchdata/GUM4dd15bfb3d0c4 319963f4662b38566e8?r=researchdata&ps=20&tab=&title=Dane% 2Bbadawcze%2B%25E2%2580%2593%2BDane%2Bbadawcze%2B% 25E2%2580%2593%2BGda%25C5%2584ski%2BUniwersytet%2BM edyczny&lang=pl&pn=1&cid=159239.
